# Clinical Performance of Nanopore Targeted Sequencing for Diagnosing Infectious Diseases

**DOI:** 10.1128/spectrum.00270-22

**Published:** 2022-03-30

**Authors:** Yu Fu, Qingsong Chen, Mengyuan Xiong, Jin Zhao, Shucheng Shen, Liangjun Chen, Yunbao Pan, Zhiqiang Li, Yirong Li

**Affiliations:** a Department of Clinical Laboratory, Zhongnan Hospital of Wuhan University, Wuhan, People’s Republic of China; b Department of Neurosurgery, Zhongnan Hospital of Wuhan University, Wuhan, People’s Republic of China; c Wuhan Research Center for Infectious Diseases and Cancer, Chinese Academy of Medical Sciences, Wuhan, People’s Republic of China; d Hubei Engineering Center for Infectious Disease Prevention, Control and Treatment, Wuhan, People’s Republic of China; University of Sussex

**Keywords:** clinical performance, infectious diseases, nanopore targeted sequencing, polymerase chain reaction, sanger sequencing, sensitivity

## Abstract

The gold standard for confirming bacterial infections is culture-positive, which has a long sample-to-result turnaround time and poor sensitivity for unculturable and fastidious pathogens; therefore, it is hard to guide early, targeted antimicrobial therapy and reduce overuse of broad-spectrum antibiotics. Nanopore targeted sequencing (NTS) is reported to be advantageous in detection speed and range over culture in prior published reports. However, investigation of the clinical performance of NTS is deficient at present. Thus, we assessed the feasibility of NTS for the first time with cohort and systematic comparisons with traditional culture assays and PCR followed by Sanger sequencing. This retrospective study was performed on 472 samples, including 6 specimen types from 436 patients, to evaluate the clinical performance of NTS designed for identifying the microbial composition of various infections. Of these samples, 86.7% were found to be NTS positive, which was significantly higher than culture-positive (26.7%). A total of 425 significant human opportunistic bacteria and fungi detected by NTS were selected to go through validation with PCR followed by Sanger sequencing. The average accuracy rate was 85.2% (maximum 100% created by Cryptococcus neoformans, the last one 66.7% provided by both Staphylococcus haemolyticus and Moraxella osloensis, minimum 0% produced by Burkholderia cepacia). The accuracy rate also varied with sample type; the highest accuracy rate was found in pleural and ascites fluid (95.8%) followed by bronchoalveolar lavage fluid (88.7%), urine (86.8%), and wound secretions (85.0%), while the lowest was present in cerebrospinal fluid (58.8%). NTS had a diagnostic sensitivity of 94.5% and specificity of 31.8%. The positive and negative predictive values of NTS were 79.9% and 66.7%, respectively. For diagnosis of infectious diseases, the sensitivity was greatly increased by 56.7% in NTS compared with culture (94.5% vs 37.8%). Therefore, NTS can accurately detect the causative pathogens in infectious samples, particularly in pleural and ascites fluid, bronchoalveolar lavage fluid, urine, and wound secretions, with a short turnaround time of 8–14 h, and might innovatively contribute to personalizing antibiotic treatments for individuals with standardized protocols in clinical practices.

**IMPORTANCE** Nanopore targeted sequencing (NTS) is reported to be advantageous in detection speed and range over culture in prior published reports. Investigation of the clinical performance of NTS is deficient at present. In our study, cohort and systematic comparisons among three assays (culture, NTS, and Sanger sequencing) were analyzed retrospectively for the first time. We found that NTS undoubtedly has incomparable advantages in accurately detecting the causative pathogens in infectious samples, particularly in pleural and ascites fluid, bronchoalveolar lavage fluid, urine, and wound secretions, with a short turnaround time of 8–14 h. For sterile specimens like blood and cerebrospinal fluid (CSF), the NTS outcomes should be validated using other nucleic acid based detection technology. Overall, NTS might innovatively contribute to guiding early, targeted antimicrobial therapy with lower cost and reduce overuse of broad-spectrum antibiotics.

## INTRODUCTION

Infectious diseases are an important cause of mortality and morbidity worldwide caused by various etiological agents including bacteria, viruses, fungi, and other microorganisms (1, 2). It is undeniable that these pathogen infections have put an immense burden on global public health systems and economies, disproportionately affecting vulnerable populations (3, 4). The crucial step of infectious diagnosis is pathogen identification by culture, which is too slow with typical turnaround times of 48–72 h (5, 6). Other limitations of culture include extremely prolonged assay times for fastidious pathogens (up to several weeks); requirements for additional testing and wait times for discerning detected pathogens (strain, virulence factors, and antimicrobial resistance); diminished test sensitivity for patients who have received antibiotics; and inability to culture certain pathogens in disease states associated with microbial infection (7, 8). Therefore, it is unable to meet the needs of timely and tailored treatments, and the reduction of the overuse of broad-spectrum antibiotics for infected patients (9). Molecular diagnostics may overcome the limitations of culture and offer the possibility of rapid reporting and improvement of the impact of clinical microbiology on patient management (10, 11).

PCR assay has been the gold standard in nucleic acid based microbial detection and diagnostics due to its ease of use, widespread instrument availability, and relatively low cost (12). However, PCR assays are predominantly singleplex, representing one reaction targeted on one marked gene for the single pathogen, found in one clinical sample (13). By contrast, unbiased multiplex metagenomic next-generation sequencing (NGS) can cover nearly all pathogens present, but its sensitivity is critically dependent on the level of background and prone to be contaminated by environmental species (14, 15). Unbiased NGS with a short-read length (50–300 bp) has a slow turnaround time and high costs because of deep sequencing and extensive bioinformatics processing, which has impeded the introduction into clinical practice (16–18). The nanopore targeted sequencing (NTS) method has the potential to overcome the shortcomings of both PCR and NGS. It is based on the combination of the long-read length (>5,000 bp) and targeted amplification (16S RNA gene for bacteria, *rpoB* for mycobacterium and ITS for fungi), which is free from the interference of host background DNA (19–23). Moreover, NTS has the advantage of rapid library preparation, real-time data acquisition, and end-to-end sequencing (19, 24). Therefore, nucleic acids with different sizes can be pooled together and sequenced from end to end in nanopore sequencing with real-time analysis (25, 26).

Previously clinical application in detecting bacterial and fungal pathogens from clinical samples was mainly focused on nanopore metagenomic sequencing, which is characterized by relatively high costs and extensive bioinformatics processing (9, 16, 27). NTS, which amplified marker genes and used a nanopore sequencing platform to sequence the amplified products, has been developed for the diagnosis of bacterial or fungal infection with fewer costs and bioinformatics processing. But the clinical performance of NTS with cohort and systematic comparisons with traditional culture assays and PCR followed by Sanger sequencing has not been investigated. Here, we assessed the feasibility of NTS by comparison with culture and the PCR followed by Sanger sequencing.

## RESULTS

### Demographic features.

A total of 472 specimens from 436 patients in Zhongnan Hospital of Wuhan University were collected. Infectious diagnosis was made strongly based on the integration of culture results and clinical presentations. The demographic description of specimens and patients with different age and gender is shown in Table 1. These patients were retrospectively classified into three major groups based on the medical records: the infectious disease (ID) (307/436, 70.4%), noninfectious disease (NID) (107/436, 24.5%,), and unknown etiology (UE) (22/436, 5.0%) groups. In the ID group, most patients were diagnosed with lower respiratory tract infections (137/307, 44.6%), followed by urinary tract infections (62/307, 20.2%), bloodstream infections (42/307, 13.7%), etc., as seen in Table 1.

**TABLE 1:** Demographic feature

Feature	Distribution, *n* (%)
Age (yr)	
14–24	13 (3.0%)
25–34	39 (8.9%)
35–44	24 (5.5%)
45–54	86 (19.7%)
55–64	110 (25.2%)
65–74	102 (23.4%)
75–84	41 (9.4%)
85–94	20 (4.6%)
95+	1 (0.2%)
Mean value (range)	58 (14–96)
Gender, *n* (%)	
Male	266 (61.0%)
Female	170 (39.0%)
Clinical diagnosis, *n* (%)	
Non-infectious disease (NID)	107 (24.5%)
Unknown etiology (UE)	22 (5.0%)
Infectious disease (ID)	307 (70.4%)
Intra-abdominal infection	24 (7.8%)
Liver abscess	2 (0.7%)
Septic arthritis	2 (0.7%)
Central nervous system infection	17 (5.5%)
Urinary tract infection	62 (20.2%)
Skin and soft tissue infection	7 (2.3%)
Upper respiratory tract infection	1 (0.3%)
Lower respiratory tract infection	137 (44.6%)
Kidney abscess	1 (0.3%)
Blood infection	42 (13.7%)
Multifocal infection	7 (2.3%)
Specimen Source, n (%)	
Bronchoalveolar lavage fluid	160 (33.9%)
Blood	121 (25.6%)
Urine	74 (15.7%)
Pleural and ascitic fluid	50 (10.6%)
Cerebrospinal fluid	42 (8.9%)
Wound drainage	25 (5.3%)
Total	472

### Pilot methods comparison based on Sanger validation.

**(i) Sample-type level.** Our results showed that pathogens determined by nested PCR were broadly in line with infectious agents uncovered by NTS from sample-type level in Table 2. Using designed specific primer pairs targeted on pathogens, 362 (85.2%) of 425 NTS-positive pathogens were verified by Sanger sequencing. What’s more, the concordance rates changed in diverse specimen types. The top two specimen types that could be demonstrated by Sanger sequencing were hydrothorax and ascites (95.8%, 46/48) and bronchoalveolar lavage fluid (BALF; 88.7%, 180/203), while CSF type was the most difficult one to be verified by Sanger sequencing with a conformation rate of 58.8% (10/17). Whereas in BALF-123, BALF-124, and BALF-129, Sanger sequencing failed to prove the existence of A. baumannii, K. pneumoniae, and S. maltophilia, respectively, with both positive NTS and culture results. NTS demonstrated C. parapsilosis existed in Urine-13 with unverified Sanger sequencing, which was also in disagreement with culture outcome (S. aureus) in Table S3 in the supplemental material.

**TABLE 2:** The comparison of three assays in different specimen types

Sample type^*a*^	The positive rate of culture (%)	No. of strains detected by culture	No. of strains confirmed by NTS	No. of strains detected by NTS	No. of strains confirmed by culture	The positive rate of NTS (%)	Sanger validation (+)	Sanger validation (–)	Total	Concordance rate
BALF	60 (37.5)	65	62 (95.4%)	238	65 (27.3%)	147 (91.9)	180	23	203	88.70%
Blood	8 (6.6)	8	5 (62.5%)	103	8 (7.8%)	91 (75.2)	43	18	61	70.50%
Urine	31 (41.9)	35	30 (85.7%)	90	33 (36.7%)	70 (94.6)	66	10	76	86.80%
Pleural and ascitic fluid	19 (38)	21	17 (81.0%)	73	21 (28.8%)	48 (96.0)	46	2	48	95.80%
CSF	4 (9.5)	4	4 (100%)	38	4 (10.5%)	31 (73.8)	10	7	17	58.80%
Wound drainage	5 (20)	6	6 (100%)	30	6 (20.0%)	22 (88)	17	3	20	85.00%
Total	127 (26.9)	139	124 (89.2%)	572	137 (24.0%)	409 (86.7)	362	63	425	85.20%

a BALF, bronchoalveolar lavage fluid; CSF, cerebrospinal fluid.

**(ii) Species-type level.** The diagnostic performance of Sanger sequencing differentiating clinically significant species (15 and 4 types for bacteria and fungi species, respectively) identified by NTS was analyzed. From the species level, the positive rates validated by Sanger sequencing are displayed in Table 3. E. coli (64/67, 95.5%), S. aureus (20/22, 90.9%), and E. faecium (18/20, 90.0%) were the top three bacterial species that could be demonstrated by Sanger sequencing. The top two fungi species proved by Sanger sequencing were C. neoformans (6/6, 100%) and C. albicans (42/45, 93.3%). The validation rates for most of the species were in the range of 80.0% to 95.5%, which met our expectations, whereas, B. cepacia (0/6) was found to be the species with the lowest positive rate confirmed by PCR testing.

**TABLE 3:** The comparison of three assays in different pathogens detected

Pathogen identifed by NTS	Sanger validation (+)	Sanger validation (–)	Positive rates	No. detected by NTS	No. proven by culture	Confirmation rate of culture	Discrepancy rate
C. neoformans	6	0	100.0%	6	3	50.0%	50.0%
E. coli	64	3	95.5%	67	42	62.7%	37.3%
C. albicans	42	3	93.3%	45	39	86.7%	13.3%
S. aureus	20	2	90.9%	22	15	68.2%	31.8%
E. faecium	18	2	90.0%	20	18	90.0%	10.0%
A. baumannii	18	2	90.0%	20	20	100.0%	0.0%
C. tropicalis	9	1	90.0%	10	10	100.0%	0.0%
E. faecalis	26	4	86.7%	30	21	70.0%	30.0%
H. parainfluenzae	27	5	84.4%	32	22	68.8%	31.3%
P. aeruginosa	37	7	84.1%	44	31	70.5%	29.5%
S. pneumoniae	15	3	83.3%	18	11	61.1%	38.9%
M. tuberculosis	5	1	83.3%	6	1	16.7%	83.3%
K. pneumoniae	36	8	81.8%	44	33	75.0%	25.0%
H. influenzae	8	2	80.0%	10	9	90.0%	10.0%
S. maltophilia	5	2	71.4%	7	7	100.0%	0.0%
C. parapsilosis	16	7	69.6%	23	14	60.9%	39.1%
S. haemolyticus	8	4	66.7%	12	9	75.0%	25.0%
M. osloensis	2	1	66.7%	3	2	66.7%	33.3%
B. cepacia	0	6	0.0%	6	4	66.7%	33.3%
Total	362	63	85.2%	425	311	73.2%	26.8%

### The comparison between NTS and culture.

**(i) The analysis of diagnostic efficacy between culture and NTS in ID and NID groups.** Based on the retrospective diagnosis of the corresponding patients, attention was especially paid to all infected and noninfected cases to analyze the diagnostic performance of NTS in Table 4. Two hundred ninety out of 307 (94.5%) patients with infectious diseases could be diagnosed by NTS, while routine culture determined 116 out of 307 (37.8%) infectious diagnosis. In the NID group, 73 out of 107 cases with noninfectious diagnosis presented positive in NTS; by comparison with using culture, only 9 noninfected cases were positive. Sensitivity was greatly increased by 56.7% in NTS compared with culture (94.5% vs 37.8%; *P* < 0.01), while specificity was higher in culture testing (91.6%) than in NTS (31.8%). Meanwhile, the positive predictive value in NTS and culture were 79.9% and 92.8% respectively. However, the negative predictive value of NTS was significantly lower than that of culture (66.7% vs 33.9%, *P* < 0.01) (Table 4).

**TABLE 4:** The diagnostic performance of NTS and culture distinguishing ID and NID

Method	Outcome	ID	NID	Sensitivity	Specificity	PPV	NPV
NTS	Positive	290	73	94.50%	31.80%	79.90%	66.70%
	Negative	17	34				
Culture	Positive	116	9	37.80%	91.60%	92.80%	33.90%
	Negative	191	98				

**FIG 1:**
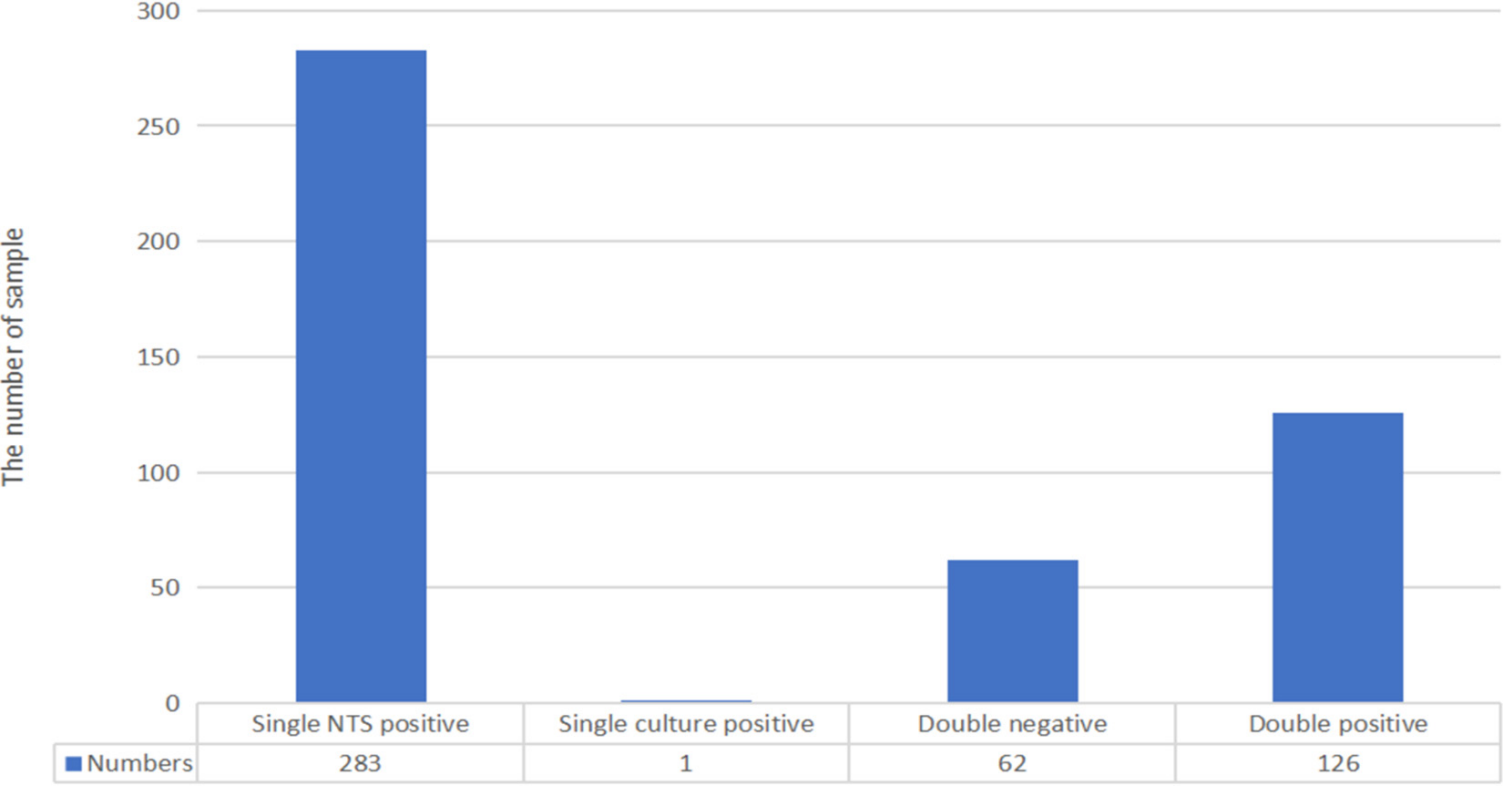
The concordance analysis of NTS and culture results. Four subsets of the combined results by both culture and NTS for all samples.

**FIG 2:**
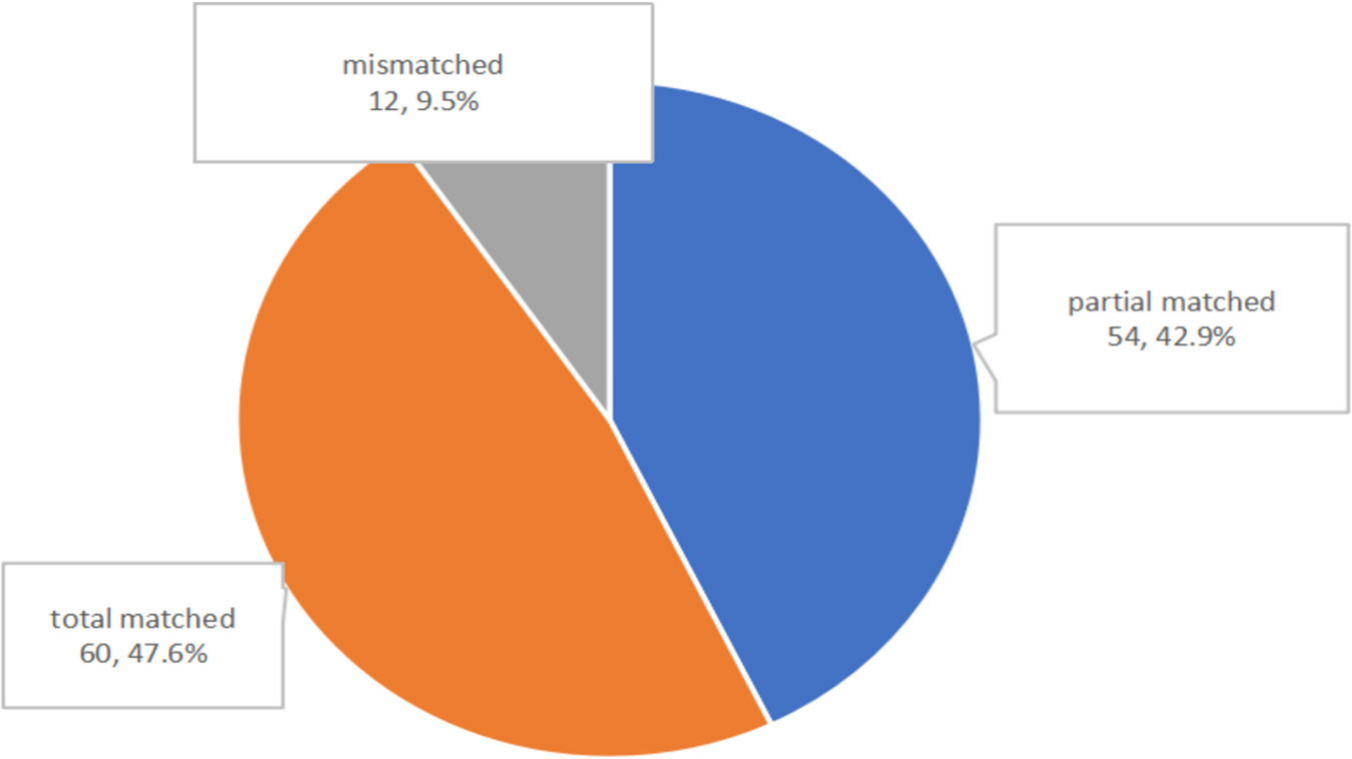
The pie chart indicated that for the double-positive subset, complete matching (60/126, 47.6%) and partial matching (at least 1 pathogen reported by NTS was confirmed in culture assays) (54/126, 42.9%) were seen, with only 12 conflicts (9.5%) between NTS and culture outcomes.

**(ii) The comparison of concordance analysis between NTS and culture.** In this study, there were four subsets of concordance analysis between NTS and culture including double-negative (62/472, 13.1%%), double-positive (126/472, 26.7%), single NTS positive (283/472, 60.0%), and single culture positive (1/472, 0.2%) (Fig. 1). Especially, the double-positive group was carefully evaluated (Fig. 2). There were 60 out of 126 (47.6%) cases of which the NTS results were totally matched to culture outcomes including both monomicrobial and polymicrobial infections; 42.9% (54/126) cases of culture outcomes partly overlapped with pathogens detected by NTS when polymicrobial infections were observed. The remaining 12 (9.5%) cases were found to be totally mismatched with the culture testing, indicative of no overlap between the two assays. What is more, among 127 culture-positive cases in ID group, NTS detected pathogens in 99.2% of cases (*n* = 126/127).

In the mismatched part of the double-positive group, NTS data of 12 specimens that were paradoxical with routine culture assays, were specifically analyzed. Additionally, the pathogens identified by NTS were utterly contradictory to those cultured from the point of genus level in 9 samples (Table S3), whereas there were three specimens in which microorganisms revealed both by NTS and culture belonged to the same family. For instance, in Urine-24, *S. haemolyticus* was identified by NTS, yet culture testing determined Staphylococcus *squirrel* as the microorganism present in the sample.

Similarly, in HA-27, both A. denitrificans (by NTS) and A. xylosoxidans (by culture) pertained to the Achromobacter genus. What’s more, in sample HA-29, culture testing determined E. faecium as the etiology, while under the same conditions, NTS confirmed E. hirae.

In the partially matched part of the double-positive subset, the pathogenic etiology reported by culture was detected together with at least one additional pathogen (not reported by culture) in 54 samples (Table S3). Potentially pathogenic organisms were also observed in 268 samples reported as NRF (normal respiratory flora)/NSG (no significant growth)/NPG (no pathogen growth) by routine microbiology. Only one conditional pathogenic organism revealed by routine culture was not detected using NTS technology, that is, Urine-18. This was reported as a noninfection with E. faecalis and E. coli, whereas only E. faecalis was detected by NTS.

In the fully matched part of the double-positive group, both monomicrobial (53/60,88.3%) and polymicrobial infections (7/60, 11.7%) were likewise detected in all 60 samples using NTS method.

In the single NTS-positive subgroup, 179 (58.3%) infectious diagnoses were assisted by NTS for patients with inconclusive results by conventional culture. Among urine specimens, infectious diagnoses were made for 25 out of 62 (40.3%) urinary tract system infected patients with negative results by conventional culture testing. There were 53 out of 121 (43.8%) blood samples confirmed by NTS data without identifiable etiology by culture. Of patients with respiratory tract infections, 43.8% (60/137) presented positive NTS results and negative culture results. Fourteen out of 17 (82.4%) patients of all central nervous system infections were diagnosed as intracranial infections on the basis of sequencing results and infectious markers in CSF. Sixteen patients were confirmed with abdominal infections with inconclusive culture results from hydrothorax and ascites samples. Eleven patients with wound infections failed to grow an isolate on culture medium.

## DISCUSSION

In this study, NTS positive rate was found to be significantly higher than culture-positive rate. The sensitivity for diagnosis of infectious diseases was increased significantly in NTS than traditional culture. Other studies have reported similar results. For example, Huang et al. found that NTS identified microorganisms in 17 of 18 aqueous humor or vitreous fluid specimens, which included 8 culture-positive specimens, 9 culture-negative specimens, and 1 specimen unavailable for culture (28). In the study by Wang et al., NTS detected pathogens in 58.7% of specimens from patients, compared to 22.1% detected using the culture method (17). We speculate that there are several reasons for such a wide variation in sensitivity between culture and NTS. The biggest one is that pathogens could be more frequently covered by empirical antibiotic usage in conventional culture testing, whereas NTS is much less affected by antibiotics (29, 30). Some fastidious bacteria such as S. pneumoniae and *M. osloensis* are difficult to grow in the medium. These are detected easily by NTS. Other slow-growing pathogens such as M. tuberculosis and fungus including C. neoformans and C. parapsilosis were often reported to be negative by culture. Thus, NTS is playing an important role for diagnosis of infectious diseases, particularly in posttreatment with empirical antibiotics, or infections caused by fastidious or slow-growing microorganisms.

PCR followed by Sanger sequencing was generally employed to validate the presence of nucleic acid of NTS or NGS-positive microorganisms. In the present study, 85.2% NTS-positive strains were verified successfully using PCR followed by Sanger sequencing. The accuracy rate was 95.8%, 88.7%, 86.8%, 85.0%, 70.5%, and 58.8% in hydrothorax and ascites, BALF, urine, wound secretions, blood, and CSF, respectively, which indicates that NTS can accurately identify most of the causative pathogens in infectious samples of hydrothorax and ascites, BALF, urine, and wound secretions. As indicated in most published references, blood and CSF were thought to be sterile body fluids, and moreover, low bacterial burden was found to be present in these two types when infection occurred. It is reasonably assumed that low bacterial burden was key for Sanger validation failures. Among the 63 unverified pathogens, 42 (66.7%) were found to be less than 400 mapped reads determined by NTS in present the study; in addition, the last three accuracy rates were found in S. haemolyticus, M. osloensis and B. cepacia. Of them, 90.9% (10/11) unverified bacteria had a burden of less than 400 mapped reads determined by NTS. Our study indicated that NTS had a higher sensitivity than PCR followed by Sanger sequencing. Supporting our results, previous study found that the NTS approach could be approximately 100 times more sensitive than real-time PCR assay (13). What’s more, high level of human background noise in blood samples that contain abundant human cells would mask the microbial sequences of interest and lead to low sequencing quality and validation failure, which was found in NGS assay (30, 31). In addition, in BALF-123, BALF-124, and BALF-129, PCR followed by Sanger sequencing failed to demonstrate the presence of A. baumannii, K. pneumoniae, or S. maltophilia with both positive NTS and culture results. Because most of these bacteria determined by NTS were successfully validated by PCR followed by Sanger sequencing, it is suggested that the nucleic acid variation of species rather than improper primers design or PCR condition may also be accountable for false negative Sanger outcomes as well as low loads of microorganisms and high background interference in collected specimens. Therefore, the actual accuracy rate of NTS assay is higher than validation rate provided by PCR followed by Sanger sequencing. In brief, NTS can accurately detect the causative pathogens in infectious samples, particularly in the hydrothorax, ascites and bronchoalveolar lavage fluid, urine, and wound secretions. In this study, the turnaround time of NTS is about 8–14 h from sample preparation to final report, which is much shorter than that of culture (48–72 h) (32) and NGS (around 4 working days) (32, 33). All in all, NTS has a very high cost–benefit ratio for diagnosis of infectious diseases featured by targeted sequencing compared with other sequencing technologies.

However, NTS still has some disadvantages due to its targeted nucleic acid amplification. There might be potential contamination generated during sample preparation and delivery. Therefore, sample quality should be strictly controlled and repeated specimens from one patient are necessary to be sent for testing in some situations. Other studies reported that targeting the conserved genes mentioned above was at times insufficient for identification of taxa at the genus level or above (34). In addition, NTS could not generate data about clinical epidemiological information including strain typing, virulence, drug-resistance genes, etc. (22). In this regard, multitargeted nanopore sequencing can be a promising tool designed to uncover species variants and emerging pathogens, discriminate closely related members of certain taxa, and reveal clinical epidemiological information including strain typing, virulence, and drug-resistance genes.

### Conclusion.

Through cohort and systematic comparisons with traditional culture assays and PCR followed by Sanger sequencing retrospectively, it was found that NTS undoubtedly has incomparable advantages in accurately detecting the causative pathogens in infectious samples, particularly in pleural and ascites fluid, bronchoalveolar lavage fluid, and urine and wound secretions, with short turnaround time of 8–14 h. For sterile specimens like blood and CSF, the NTS outcomes should be validated using other nucleic acid based detection technology. Overall, NTS might innovatively contribute to guiding early, targeted antimicrobial therapy and reduce overuse of broad-spectrum antibiotics.

## MATERIALS AND METHODS

### Study patients and sample collection.

In this retrospective study, 472 residual clinical specimens from 436 patients in Zhongnan Hospital of Wuhan University were collected and sent for pathogen identification by NTS and culture simultaneously from March 2021 to May 2021. All of the samples were classified into 6 categories, including 160 bronchoalveolar lavage fluid (BALF), 121 EDTA-whole blood, 74 urine, 50 pleural and ascites fluid, 42 cerebrospinal fluid (CSF), and 25 wound secretions (Table 1). Four hundred thirty-six clinical medical records were retrieved and then assessed. All study patients were classified into three major groups as noninfectious disease (NID), infectious disease (ID), and the unknown group with unexplained etiology according to medical records documented by clinicians. In the ID group, patients were retrospectively diagnosed with various types of infections presented in Table 1. All clinical diagnosis were made carefully based on orthogonal regular laboratory tests including culture, specific antibody, and antigen testing. In addition, clinical presentations were undoubtedly taken into account that involved patients’ symptoms (fever, cough, dizziness, nausea, etc.) and related infectious markers including white blood cells count (WBC), C-reactive protein (CRP), procalcitonin (PCT), and interleukin-6 (IL-6).

### Sample processing and nucleic acid extraction.

All samples were collected in sterile tubes and sent to the clinical laboratory for DNA extraction. Specific pretreatment procedures were performed according to different sample types. Briefly, all liquid samples except blood were centrifuged at 20,000 × *g* for 10 min. The supernatant was removed and 200 μL of the sample was retained for DNA extraction. As for blood, 1.5 mL of EDTA-whole blood samples were centrifuged at 800 × *g* for 10 min at room temperature. Then, the lower part of red blood cells was discarded and about 600 μL of supernatant including leukocytes and plasma were separated and transferred to a new Eppendorf tube, and were centrifuged at 16,000 × *g* for 10 min; the supernatant was discarded and 200 μL of precipitate was removed for subsequent DNA extraction. Wound secretions were collected by sterile swabs. Swabs were vortexed in 1 mL sterile saline and centrifuged at 20,000 × *g* for 10 min. The supernatant was removed, and 200 μL of saline was added for DNA extraction. DNA was extracted from 200 μL of pretreated samples within 6 h, using the Sansure DNA Extraction Kit (Changsha, China) following the manufacturer’s instructions, which involved thermal disruption (95°C for 15 min) to allow for the release of nucleic acids from hard-to-lyse organisms. TE buffer of each batch was examined as the negative control for DNA extraction.

### Amplification and nanopore targeted sequencing.

NTS was built by targeted amplification of the 16s rRNA gene (for bacteria), IST1/2 gene (for fungal), and rpoB (for Mycobacterium spp.) using universal and specific primers, and sequenced by a real-time nanopore sequencing platform. The 27F/1492R and ITS1/4 primers were employed as the start primers for amplification of bacterial 16S rRNA and fungal internal transcribed spacer regions 1 and 2 (ITS1/2), respectively; the additional primers with barcode are also listed in Table S2, which would make organisms amplified and sequenced successfully by reducing the risk of amplification failure created by variation of each base (35–38). All the primers should meet the following standards: (i) 18–30 bp for primer length; (ii) melting temperature (Tm): 58–65°C, with a temperature difference of less than 3°C between start tube and additional primer; (iii) GC content of primers: 40–60%; (iv) ΔG (Gibbs free energy) of the last five resides of the primers at the 3′ end: ≥–9 kcal/mol (17). The 27F/1492R or ITS1/4 primer and its additional primers was blended with the molar ratio of 3:1 for amplification. For Mycobacterial *rpoB*, MF/MR primer and the additional primers were especially mixed with the molar ratio of 3:1 to obtain the final specific primer pairs listed in Table S2. The full lengths of 16S rRNA, ITS, and *rpoB* are about 1.5 kb, 400–800 bp, and about 400 bp in this study. Amplification of the 16S rRNA gene and *rpoB* were performed in a 20 μL reaction system with 8 μL extracted DNA, 2 μL barcoded primer consisting of random N bases (10 μM), and 10 μL 2×KOD OneTM PCR Master Mix (TOYOBO) using the following procedure: 1 cycle at 98°C for 3 min, 35 cycles at 98°C for 10 s, 55°C for 5 s, and 68°C for 10 s, followed by a final elongation step at 68°C for 5 min. ITS1/2 was firstly amplified using the same reaction system, and PCR procedure was performed using the primer for ITS1/2 without a barcode (1 cycle at 98°C for 3 min, 35 cycles at 98°C for 10 s, 55°C for 5 s, and 68°C for 10 s, followed by a final elongation step at 68°C for 5 min); the PCR product was purified using 0.8× AMpure beads (Beckman Coulter) and eluted in 10 μL Tris-EDTA buffer. Then, 5 μL of the eluate was used for the barcoded PCR with 5 μL of the barcoded ITS1/2 primer set (10 μM) and 10 μL 2× Phusion U Multiplex PCR Master Mix using the following procedure: 1 cycle at 98°C for 3 min, 10 cycles at 98°C for 10 s, 55°C for 5 s, and 68°C for 5 s, followed by a final elongation step at 68°C for 5 min. The barcoded amplification products of the 16S rRNA gene, ITS1/2, and *rpoB* from the same samples were pooled at a mass ratio of 10:3:1. The pooled products from the different samples were equally mixed and used to construct sequencing libraries using the 1D Ligation Kit (SQK-LSK109; Oxford Nanopore). Clinical samples and two Tris-EDTA buffers (no-template control, NTC) were batched in one sequencing library, and the library was sequenced using Oxford Nanopore GridION X5 with real-time base calling enabled (ont-guppy-for-gridion v. 1.4.3-1 and v. 3.0.3-1; high-accuracy base calling mode) (39).

### Standard strains and mock communities.

The evaluation of performance by NTS was based on the standard strains and mock community as the positive controls. The standard strains were purchased from the American Type Culture Collection (ATCC) and could be separately identified correctly by NTS at the species level. Mock communities were composed of three bacteria and three fungi (Moraxella catarrhalis, Acinetobacter baumannii, Staphylococcus aureus, Candida glabrata, Candida parapsilosis, Candida tropicalis). They were constructed to reproduce a complex clinical specimen, and the proportion of each strain was changed and determined by calibration curves from cultures.

### Bioinformatics analysis pipeline.

Base-calling and quality assessment of sequencing data were performed using Oxford Nanopore GridION X5 and Guppy in high accuracy mode (ont-guppy-for-gridion v. 1.4.3-1 and v. 3.0.3-1; high-accuracy base calling mode). Sequencing reads with low quality (Q score <7) and undesired length (<200 nt or >2000 nt) were discarded. An in-house script was used to analyze the output of the base calling data and generate a real-time taxonomy list of each sample by screening and starting the bioinformatic pipeline when every 4,000 reads passed the base calling process. Briefly, Porechop (v. 0.2.4) was used for adaptor trimming and barcode demultiplexing for retained reads that passed the basecalling process. The reads of each sample were mapped against the 16S rDNA/ITS reference database collected from NCBI FTP (ftp://ftp.ncbi.nlm.nih.gov/refseq/TargetedLoci) using BLAST. Reads with alignments that exhibited both >80% identity and >80% query coverage were retained (40). Then, the taxonomy of each read was assigned according to the taxonomic information of the mapped subject sequence. For the reads preliminarily assigned to the same species, a consensus sequence was generated using Medaka (v. 0.10.1). Then, the consensus sequence was remapped to the 16S rDNA/ITS reference database, and the best-assigned taxon was used as the final detection result of reads from the same species of the preliminary taxonomy assignment. The pathogen detection of the clinical sample was interpreted according to a strict set of rules as follows (17). The criteria for calling a positive result of bacterial or fungal identification by NTS is as follows: mapped reads of bacterial species in specimens >100 or is more than that of any other species, and the ratio of mapped reads in the specimen and negative control >10; mapped reads of fungi from the species level >20 or higher than 50% of relative richness, and the ratio of mapped reads in the specimen and negative control >10. There are critical lists of bacteria and fungi that have been clinically known to be typically or potentially pathogenic reported from clinical guidelines and literature (41).

**FIG 3:**
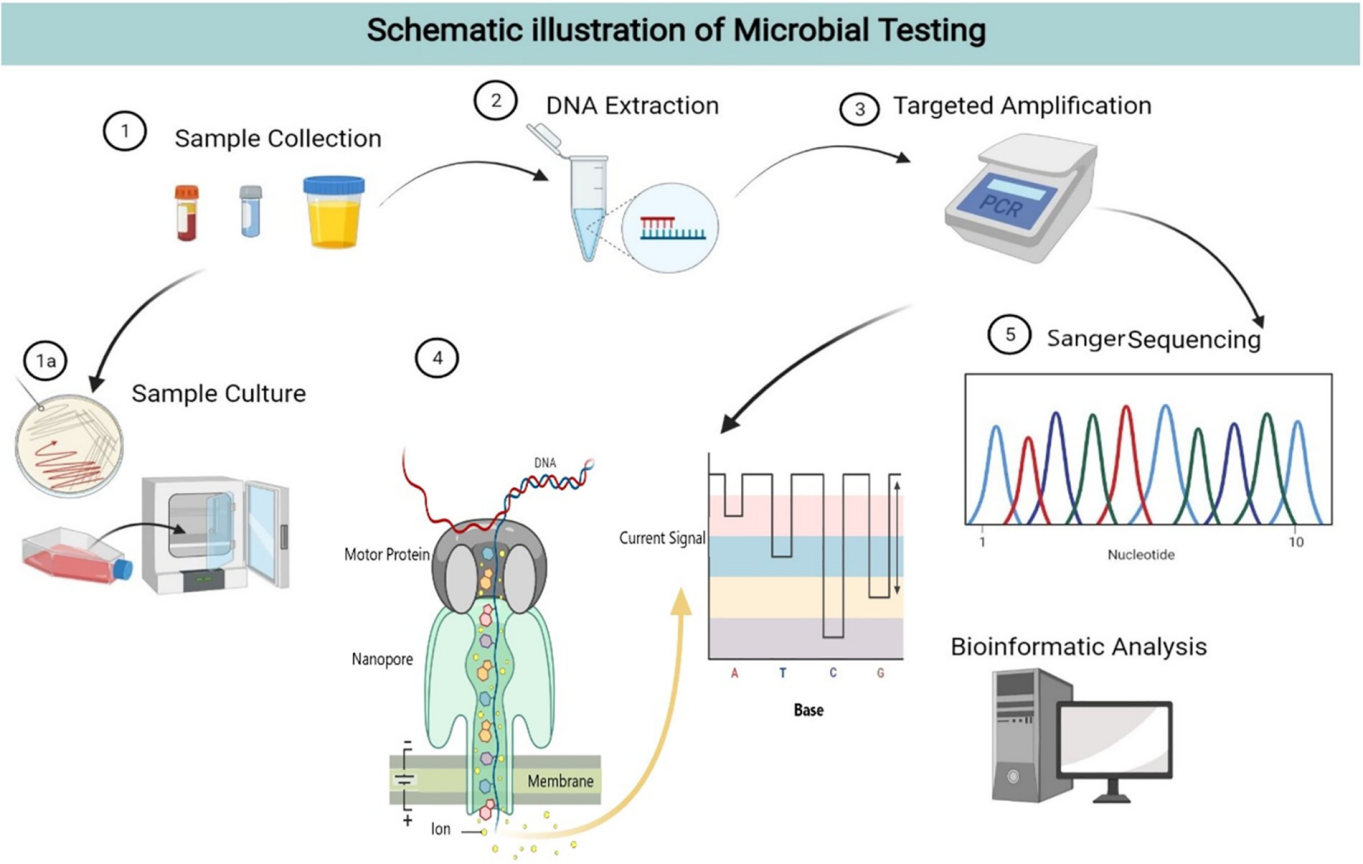
The schematic illustration of microbial testing, including sample collection and culture, DNA extraction, targeted amplification followed by nanopore targeted sequencing, and bioinformatic analysis. Meanwhile, the NTS data went through Sanger validation. This figure was created with BioRender (https://biorender.com).

Furthermore, the amplified products of specific marker genes with positive PCR results were analyzed by Sanger sequencing afterwards to identify the presence or absence of the missed/additional pathogens detected by NTS. The whole workflow of microbial testing in this study is presented in Fig. 3 (created by BioRender; https://Biorender.com).

**FIG 4:**
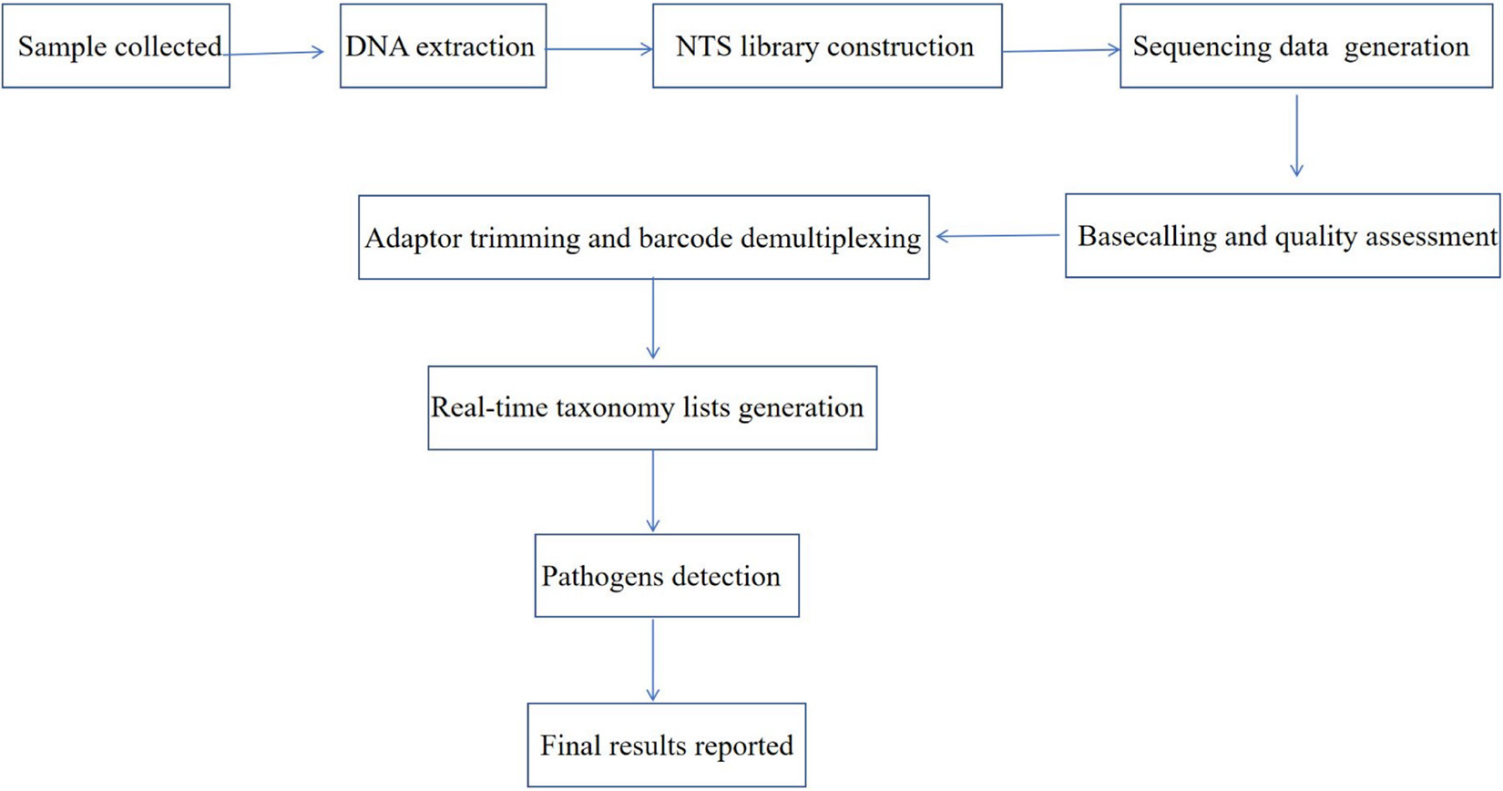
Protocol for NTS Assay. After samples are received in the clinical laboratory, nucleic acid (DNA) is extracted, followed by construction of NTS target library and sequencing. The NTS data are analyzed with the use of Oxford Nanopore GridION X5 and Guppy in high accuracy mode (ont-guppy-for-gridion v. 1.4.3-1 and v. 3.0.3-1; high-accuracy basecalling mode), An in-house script was used to analyze the output of the basecalling data and generate a real-time taxonomy list of each sample by screening, Porechop (v. 0.2.4) was used for adaptor trimming and barcode demultiplexing for retained reads that passed the basecalling process; pathogen detection of the clinical sample was interpreted and reported in the electronic medical record.

The protocol for NTS assay including bioinformatics analysis pipeline is demonstrated in Fig. 4. After samples are received in the clinical laboratory, nucleic acid (DNA) is extracted, followed by construction of NTS target library and sequencing. The NTS data are analyzed with the use of using Oxford Nanopore GridION X5 and Guppy in high accuracy mode (ont-guppy-for-gridion v. 1.4.3-1 and v. 3.0.3-1; high-accuracy basecalling mode). Then the output of the basecalling data was analyzed and a real-time taxonomy list of each sample was generated by screening; Porechop (v. 0.2.4) was used for adaptor trimming and barcode demultiplexing for retained reads that passed the basecalling process; and pathogen detection of the clinical sample was interpreted and reported in the electronic medical record.

### Culture-based microbiological assay.

Non-sterile body fluids (urine, pleural and ascitic fluid, BALF, wound drainage) were all inoculated into blood agar and MacConkey Agar (MAC) plates, respectively. Sterile body fluid samples such as CSF and whole blood were inoculated into chocolate agar plates except blood agar plates. All culture plates were incubated at 37°C overnight for bacterial detection. For fungi identification, Sabouraud dextrose agar tubes were used and incubated at 25°C for 48–72 h in the air with 5% CO2. The interpretation of positive culture results depends on the type of specimens. For sterile body fluids like blood and CSF, any clone of isolates on the plate was interpreted as a positive result and should be reported to the clinicians immediately after excluding any potential contamination. For nonsterile clinical samples, type and quantity of strains should both be considered for determination of positive culture result. For example, the standard cutoff for a positive urine culture is >100,000 CFU/mL (42), and staphylococcus *albus* should be reported as bacterial contamination. Bacterial and fungal identification was done from positive culture plates using standard biochemical tests and MALDI-TOF (matrix-assisted laser desorption/ionization time-of-flight mass spectrometry; Vitek 2, Biomerix, France) assay using a strain of Escherichia coli (ATCC 8739) as control.

### Nested PCR followed by Sanger validation.

To testify to the detection correctness by NTS, we performed PCR testing with residual DNA sample extracted from clinical specimens after positive NTS analysis. Considering the wide varieties of microbial communities that existed in specimens, 19 clinically significant opportunistic pathogens were selected to go through verified PCR experiment followed by Sanger sequencing. Those included bacteria (Klebsiella pneumoniae, Escherichia coli, Staphylococcus aureus, Mycobacterium tuberculosis, Acinetobacter baumannii, Pseudomonas aeruginosa, Staphylococcus haemolyticus, Stenotrophomonas maltophilia, Burkholderia cepacian, Haemophilus influenzae, Streptococcus pneumoniae, Enterococcus faecalis, Enterococcus faecium, Moraxella osloensis, Haemophilus parainfluenzae) and fungi (Cryptococcus neoformans, Candida albicans, Candida tropicalis, and Candida parapsilosis). All primers used in nested PCR are listed in Table S1, and all were synthesized by Tianyi Huiyuan Biotech Co., Ltd, Wuhan, China. All PCR mixture volume (25 μL) contained 5 μL of template DNA (1 ng–10 μg), 1 μL of each primer (10 μM), 12.5 μL 2×*Taq* PCR MasterMix (Aidlab Biotechnologies Co., Ltd), and 5.5 μL double distilled water (ddH2O). The nested PCR protocol included the first round with nested-outer primers (94°C for 3 min; 30 cycles of 94°C for 30 s; 55°C for 30 s; 72°C for 30 s; and 72°C for 5 min), the second round with nested-inner primers (94°C for 3 min; 35 cycles of 94°C for 30 s; 55°C for 30 s; 2°C for 30 s; and 72°C for 5 min). The PCR products for sequencing were cleaned up and normalized using the SequalPrep DNA Normalization Kit (Invitrogen) according to the manufacturer’s recommendations and eluted in 20 μL elution buffer. Based on the 19 clinically significant pathogens, nested PCR was performed with 425 reactions. PCR products were analyzed by agarose gel electrophoresis and purified with a DNA gel extraction kit (Simgen). Sanger sequencing was performed on an ABI PRISM 3730 DNA Sequencer (Applied Biosystems, Foster City, CA, USA) for validation. Then, sequence information was aligned with database using NCBI BLAST online software to make sure the NTS data were consistent with Sanger Sequencing (https://blast.ncbi.nlm.nih.gov/Blast.cgi).

### Data analysis.

Data were analyzed by IBM SPSS Statistics 26.0. A *P* value < 0.05 was considered significant.

### Ethics.

Ethical approval for this study was obtained from the ethics committee of Zhongnan Hospital of Wuhan University. For method development purposes, excess samples from routine microbial culture were submitted to NTS in a microbial laboratory. Informed consent was waived because it was a retrospective study and did not involve any sensitive information.
